# Sensory characterization of functional guava symbiotic petit cheese product

**DOI:** 10.1016/j.heliyon.2023.e21747

**Published:** 2023-11-07

**Authors:** Victor Iván Morales-Cortés, Julieta Domínguez-Soberanes, Linda Carolina Hernández-Lozano, Carmen C. Licon, Antonio Estevez-Rioja, Mayeli Peralta-Contreras

**Affiliations:** a Universidad Panamericana. Escuela de Dirección de Negocios Alimentarios. Jose María Escrivá de Balaguer 101, Villas Bonaterra, Aguascalientes, 20296, México; b Universidad Panamericana. Facultad de Ingeniería. Jose María Escrivá de Balaguer 101, Villas Bonaterra, Aguascalientes, 20296, México; c Dairy Products Technology Center, California Polytechnic State University, 1 Grand Avenue, Building 18A, San Luis Obispo, CA, 93407, USA

**Keywords:** Dairy products, Petit suisse, Probiotics, Prebiotics, Symbiotics, Low calorie, Sensory analysis, Sensory-driven

## Abstract

The consumption of functional dairy products continues to rise due to consumer needs. This study aimed to develop a dairy guava functional symbiotic petit cheese product that included probiotics (*Bifidobacterium animalis* subsp. *lactis* BB-12, Chr. Hansen, Denmark) and prebiotics (inulin), which had adequate organoleptic characteristics. Moreover, adequate physicochemical, microbiological, and sensory characteristics during its shelf life were expected. A pasteurized skim milk curd flavored with a guava pulp was stabilized with gelatin to formulate this product. As sweeteners, iso maltol, erythritol, and Luo Han Guo extract from monk fruit (*Siraitia Grosvenorii*) were added. The prebiotic used was inulin, and the probiotic (*Bifidobacterium animalis* subsp. *lactis* BB-12, Chr. Hansen, Denmark). The product was kept refrigerated (4 °C) during the shelf life of 28 days. For the organoleptic analysis (100 consumers), the evaluations performed were: (1) overall liking (OL), (2) CATA (Check all that apply) testing 19 attributes, and (3) purchase intention was evaluated. Results were analyzed with FIZZ Software Biosystèmes. During shelf life, (1) physicochemical, microbiological, and sensory tests were performed. The product was evaluated as “liked much'' (7.16 out of 9); it was described as a creamy (71 %) natural product (73 %) with a fruity odor (57 %). It could be suitable for marketing because 82 % of the consumers would buy it. The product's probiotic character (over 1 × 10^6^) was established through a microbiological count. On day one, the CFU was found to be 4.15 × 10^8^, and after 28 days, 1.98 × 10^8^ CFU of viable Bifidobacterium *animalis* subsp. *lactis* BB-12, leading us to establish its probiotic characteristics. The shelf life was estimated at 21 days.

## Highlights

• Consumers are looking for new healthy and nutritious products.
• Petit Suisse can be used as a functional matrix to incorporate prebiotics and probiotics.
• Low-calorie sweeteners (erythritol, iso maltol, and Luo Han Guo) are good sugar replacers in petite Suisse products.
• Inulin can proved adequate sensory characteristicas in petit Suisse products.
• Of the 100 consumers recruited for sensory evaluation, 82 % would buy the product.

## Graphical abstract

**Figure:**
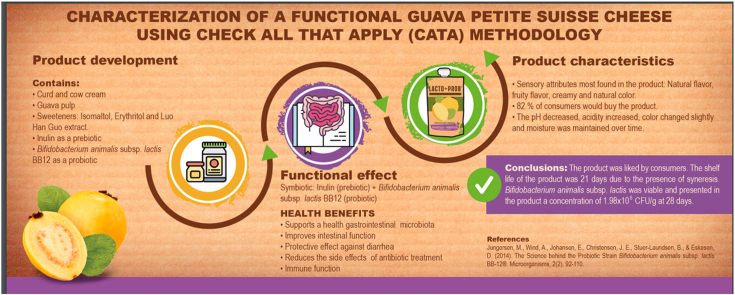

## 1 Introduction

There is a pressing need to develop foods that fulfill the body's nutritional requirements while offering health advantages. The COVID-19 pandemic has imparted several lessons, notably the significance of maintaining good health through a balanced diet. Aligned with this trend, the present research is focused on formulating a functional guava Petit Suisse cheese enhanced with *Bifidobacterium animalis* subsp. *lactis* BB-12. Extensive clinical studies have demonstrated the favorable impact of this bacterium on health. These benefits include improvement of the following matters:

(1) Gastrointestinal health and immune function [1];
(2) An increase of the presence of probiotics could contribute to the dental well-being [2];
(3) Steady improvement of glucose metabolism and insulin sensitivity during and after pregnancy [3];
(4) Efficacy in alleviating colic, resulting in reduced infant crying and irritability [4];
(5) Increased survival of BB-12 through the gastrointestinal tract [1].

Petit Suisse cheese traces its origins to France. It is believed to have been created by a Swiss cheesemaker in Normandy during the mid-19th century, hence its name. This cheese is crafted by blending curds with cream, yielding a product containing over 40 % of its weight in fat. This cheese is produced through milk coagulation using mesophilic bacteria, followed by homogenization [5], obtaining a product with a texture more likely to have sensory characteristics as a yogurt than a cheese. In Mexico, lighter versions, with lower fat content and as an unripened cheese, are available, often sweetened with sugar and fruit [6]. Petit Suisse can be described as a soft cheese consumed as a dessert or snack.

Moreover, this product is an adequate matrix for incorporating all sorts of ingredients, such as probiotics, prebiotics, and other ingredients as fruits that can have other nutritional functions such as antioxidants [7,8,9,and^10^]]. This type of cheese is widely accepted by consumers, especially by children. In addition, research [11], establishes that it could have possibilities for consumption by other market groups because of its flavor, composition, and functional appeal. Therefore, the study suggests that more research could be performed in order to evaluate the competitiveness of this kind of product on the market.

Prebiotics can be defined as a substrate that can stimulate the growth of host microorganisms, thus conferring health benefits [12,13]. Prebiotics typically comprise non-digestible carbohydrates, usually oligosaccharides and polysaccharides, which human enzymes cannot hydrolyze.

On the other hand, beneficial bacteria belonging to the genera Lactobacillus and Bifidobacterium grow in the gastrointestinal tract [14]. Furthermore, prebiotics can shield probiotics, augmenting their survival. The probiotic strain, prebiotic component, and the dairy matrix influence this survival [13].

Several benefits have been associated with prebiotics [14]:

1) Relief from constipation,
2) Increased mineral absorption,
3) Intestinal pH reduction,
4) Intestinal bacterial balance restoration, and modulation of lipid metabolism,
5) Anticarcinogenic effects
6) Impacts on the immune system

In particular, inulin compounds are integral to a non-digestible polysaccharide called fructans. Therefore, inulin is a functional component [15], which can improve gastric health and boasts techno-functional properties such as fat substitution, texture modification, and sugar replacer. Moreover, the incorporation of inulin has been tested in Petit Suisse cheeses with promising results [16].

After COVID, there is a worldwide trend for sugar reduction, with natural sweeteners; which means replacing refined sugar with alternative sweeteners due to the potential health benefits offered by other ingredients [17,18]. Refined sugar, commonly referred to as table sugar, primarily consists of fructose and glucose in the form of sucrose. Its increased consumption contributes to a high calorie intake, directly associated with health problems. The health issues are associated with cerebrovascular and cardiovascular diseases, diabetes mellitus, cancer, dental caries, obesity, and impaired cognition [17]. Luo Han Guo extract, a natural, non-nutritive sweetener associated with properties such as anti-cancer, antioxidant, anti-inflammatory, anti-obesity, and antidiabetic [18]. Other promising novel natural sweeteners are erythritol and allulose, which have proven to be a good alternative for developing new food products [19]. The integration of these sweeteners aligns with the sugar-free labeling stipulated by the Mexican Official Standard NOM-051-SCFI/SSA1-2010 [20]. Furthermore, their usage reduces calorie intake, as advocated by the World Health Organization (WHO) and the Food and Agriculture Organization of the United Nations (FAO) [21].

Guava was selected in this study due to its delightful flavor profile. In addition, it holds the distinction of being the most extensively cultivated fruit in Aguascalientes, Mexico. Its widespread availability throughout the year and its nutritional and health-promoting attributes make it one of the most consumed fruits. Numerous studies have identified bioactive compounds like phenolic acids (caffeic acid), flavonoids (quercetin and kaempferol), and carotenoids (beta-carotene and lycopene) with guava. These compounds play a pivotal role in preventing cerebrovascular, cardiovascular, and cancer-related issues, thus enhancing the functional impact of the product [22,23,24,and^25^]].

Therefore, the objective of this study aimed to develop a functional guava symbiotic petit cheese product; the food system developed included probiotics (*Bifidobacterium animalis* subsp. *lactis* BB-12, Chr. Hansen, Denmark) and prebiotics (inulin) integrated in the dairy matrix. The resulting product was monitored throughout its shelf life in its physicochemical, microbiological and sensory changes. The product development was conducted at the food technology laboratory of the Universidad Panamericana Aguascalientes, Mexico.

## 2 Methodology

### 2.1 Product elaboration

The formulation used to make Petit Suisse cheese is presented in Table 1.

**Table 1:** Guava petit Suisse cheese formulation.

Component	Quantity (kg)	Percentage (%)
Curd	1.040	48.22 %
Guava pulp	0.521	24.15 %
Cow cream 30 %	0.287	13.31 %
Isomaltol	0.150	6.95 %
Erythritol	0.092	4.27 %
*Bifidobacterium animalis* subsp. *lactis* BB-12 added with milk	0.045	2.09 %
Inulin	0.007	0.32 %
Gelatin	0.006	0.28 %
Salt	0.005	0.23 %
Luo Han Guo extract	0.002	0.09 %
Citric acid	0.002	0.09 %
Total	2.157	100.00 %

**Fig. 1:**
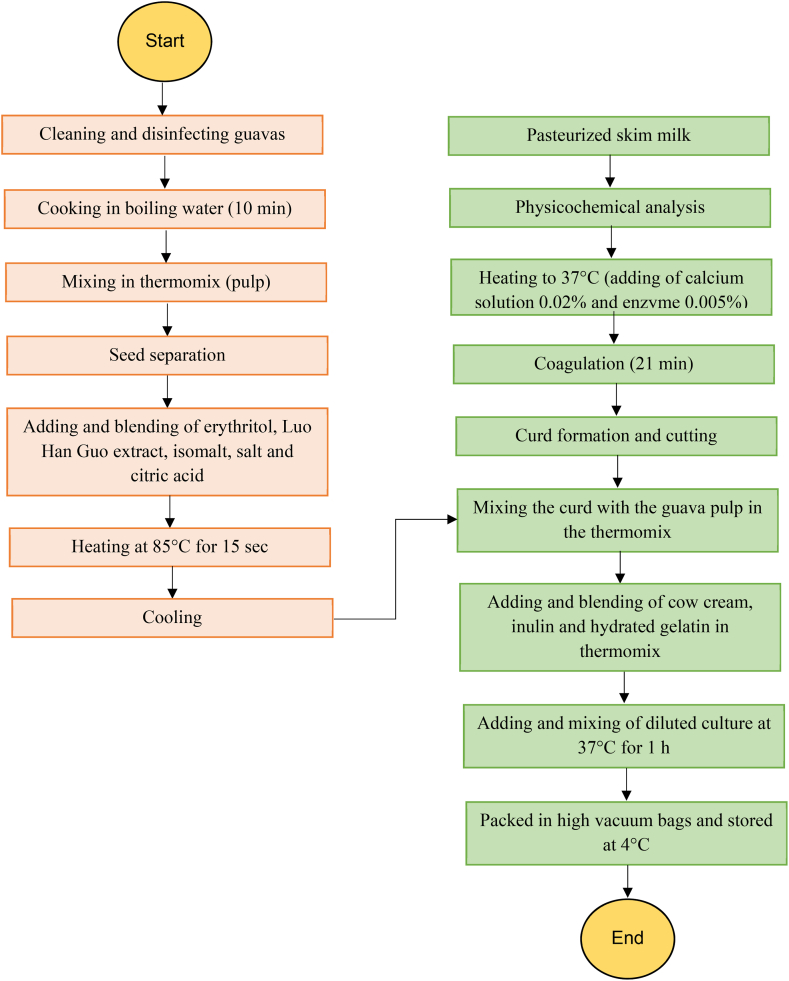
Process diagram of petit Suisse cheese.

The methodology for making guava Petit Suisse cheese is described in a process diagram in Fig. 1.

#### 2.1.1 Ingredients

##### 2.1.1.1 Pulp and sweeteners

The pulp was obtained from the fresh guava. The fruit was boiled in water for 10 min (Ferro, México). Then it was mixed in the thermomix (Vorwerk, Germany) at speed 6 for 3 min. A seed separation was performed through a stainless steel strainer, according to procedure established in Fig. 1.

Subsequently, erythritol, Luo Han Guo extract from monk fruit (*Siraitia grosvenorii*), iso maltol, salt, and citric acid were added to the pulp mixing all in the thermomix (Vorwerk, Germany) at speed 3 for 1 min (Fig. 1).

Table 1 shows the concentrations of the ingredients in the formulation.

In order to eliminate possible microbiological growth, the pulp was subjected to a thermal process at 85 °C for 15 s in the thermomix (Vorwerk, Germany). The pulp was cooled to 4 °C.

##### 2.1.1.2 Curd manufacturing

As observed in Fig. 1, curd was obtained by heating the milk at temperature of 37 °C. Subsequently, diluted calcium chloride was added at a concentration of 0.02 %. Later, rennet 1:10,000 (Cuamex, Mexico) was diluted in water at a concentration of 0.005 %. The inoculated mix was allowed to rest for 21 min, followed by cutting the curd with a stainless steel knife. Later on, the curd was cut into approximately 1-cm3 cubes. Then the curd was stirred for 5 min to facilitate dehydration. Finally, it was drained for 15 min to remove excess of whey.

##### 2.1.1.3 Prebiotic and probiotic

The prebiotic, inulin (Preventy, México), was added in a 0.32 %. On the other hand, *Bifidobacterium animalis* subsp. *lactis* BB-12 was incorporated in 2.09 %; as recommended by the supplier (Chr. Hansen, Denmark). The latter for obtaining a bacterial load higher than 1 × 10^6^ CFU/g in the final product. For which, the lyophilized *Bifidobacterium animalis* subsp. *lactis* BB-12, culture (5 g) was diluted in (40 mL) of pasteurized skimmed milk and was incubated at 37 °C for 24 h.

##### 2.1.1.4 Gelatin

The gelatin (Duche, Mexico), which had a 290 Bloom, was hydrated by diluting it with water in a 1:2 ratio. The final concentration of this mixture was 0.28 % of the total concentration of the formulation.

#### 2.1.2 Ingredient incorporation

In order to prepare the Petite Suisse cheese, all the ingredients (Table 1) were mixed together as shown in Fig. 1. Therefore, the curd, and the guava pulp were mixed in a Thermomix (Vorwerk, Germany) at speed 6 for 3 min. During this time, the 30 % cow cream was gradually added, along with the prebiotic and gelatin mixture. Finally, the probiotic culture was added to the mixture.

#### 2.1.3 Packaging and product storage

The samples were vacuum-sealed in bags and refrigerated at temperatures ranging from 0 to 4 °C for storage. Product's shelf life was evaluated.

### 2.2 Physicochemical analysis

The following analysis were performed by triplicate.

#### 2.2.1 Humidity

Humidity was determined, by triplicate, calculating weight loss due to water evaporation using the methodology described in NOM-243-SSA1-2010) [26]. The curds and the Petit Suisse cheese were analyzed for humidity during their shelf life.

#### 2.2.2 Color

Color was measured, by triplicate, using the colorimeter (Nix Pro, Canada). The measurement was performed directly on the packaging (transparent bag), placing the colorimeter on the surface. The results obtained were the L*a*b* coordinates of the CIELAB system and captured through a smartphone.

#### 2.2.3 pH

pH, which was performed in triplicate, was measured using the methodology described in NOM-F-317-S-1978 [27] with a potentiometer (Ohaus, USA). The pulp, milk, curd and the Petit Suisse were analyzed.

#### 2.2.4 Total soluble solids

Total soluble solids, which were performed using a refractometer (Sper Scientific, China). For the analysis, a mixture drop was put into the equipment for measurement. Total soluble solids were determined in the pulp by triplicate.

#### 2.2.5 Titratable acidity

Titratable acidity was performed by triplicate. It was carried out with a NaOH solution at 0.1 N. (JT Baker, US). The indicator was phenolphthalein 1 %, which was diluted in 70 % ethanol (Golden Bell, Mexico), using the methodology described in NOM-243-SSA1-2010 [26]. The milk was analyzed for titratable acidity.

#### 2.2.6 Syneresis

Syneresis was evaluated visually in the Petite Suisse on the vacuum-sealed bag over time. A subjective measure was employed to characterize the product, and the parameters were used according to a scale elaborated in the Universidad Panamericana. The scale used four parameters to describe the product: not present, slightly present, present, and excessively present.

### 2.3 Organoleptic analysis of the product

For the organoleptic analysis, 100 consumers ranging from 18 to 23 years on average, who were students from Universidad Panamericana Aguascalientes, participated in this study. All consumers agreed to participate in this research and signed an Informed Consent Form. They answered a sensory questionnaire that evaluated the sensory characteristics of the Petite Suisse.

Samples, which were previously coded with a 3-digit identifier, were served in 1-ounce plastic cups that contained 30 g of the product and were served at 6 °C.

The questionnaire was divided into three sections: (1) liking evaluation, (2) CATA Test and (3) Purchase intention.

#### 2.3.1 Liking evaluation

First, the consumers were asked to grade the acceptance of the product on a 9-point hedonic scale.

#### 2.3.2 CATA test (check all that apply)

Secondly, the CATA test (Check all that apply) that had nineteen attributes was applied to consumers. Consumers were instructed to check the attributes that they found present in the product. The attributes evaluated were: The attributes studied were: pleasant view, natural color, artificial color, pleasant smell, fruity flavor, no odor, tasteless flavor, artificial flavor, natural flavor, fruity flavor, milky flavor, lack of flavor, creamy, sweet, acidic, firm texture, fluid texture, good aftertaste and lumpy [28,29].

#### 2.3.3 Purchase intention

Finally, consumers answered whether they would buy the food product or not.

### 2.4 Shelf life study

The shelf life study was conducted in real-time for 28 days, taking samples on days 1, 7, 14, 21, and 28. The following physicochemical parameters were determined (triplicate): a) humidity, b) color, c) tiratable acidity, and d) syneresis. The methodology of these tests is explained in section 2.2.

The methodology for microbiological analysis is explained in section 2.4.1 and 2.4.1. In section 2.4.1. the methodology for total aerobic bacteria, *E. coli*, and total coliforms can be observed. On the other hand, in order to evaluate the functional effect of the product, a viable bacterial count was performed *on Bifidobacterium animalis* subsp. *lactis* BB-12 through microbiological analysis of lactic acid bacteria. The methodology is explained in section 2.4.2.

The sensory test was performed using the methodology of RATA (Rate all that apply). This sensory test was carried out to evaluate the sensory characteristics. The product was sensory evaluated on days 1, 7, and 21.

#### 2.4.1 Microbiologic analysis

The material was prepared for the microbiological analysis as follows: To 250 mL flasks (Glassco, India), 90 mL of distilled water was added. Test tubes were prepared with 9 mL of distilled water, and the micropipette was sterilized in a vertical autoclave (Novatech, México). The autoclave was heated until reaching a pressure of 1.5 kg/cm^2^ and a temperature of 120 °C. Once the pressure and temperature were achieved, they were maintained for 15 min. Next, 10 g of the sample was weighed and placed in a bag (Whirl Pak, USA), and 90 mL of sterile water was added in order to obtain a dilution of 10 ^−1^. For which, a sample volume with 1 ml of the dilution 10^−1^ was used. The microbiological analyses were performed in a laminar flow hood (Novatech, Mexico). The growth of total aerobic bacteria in was obtained in 3 M™ Aerobic Count Plates Petrifilm™. For the *E. coli,* 3 M™ *E. coli* Petrifilm™, were used. And for the coliform count, 3 M™ Coliforms 6404 Petrifilm™, were used. The incubation (Novatech, Mexico) took place under different parameters according to the type of microorganism. For aerobic total bacteria, the incubation parameters were 48 h at 35 °C ± 1. The parameters for coliforms were 24 h at 37 °C ± 1. Finally, for *E. coli* 48 h at 37 °C ± 1. The counting of viable cells and interpretation of results were carried out as suggested by the supplier of the 3 M México plate [30,31]. The microbiological study was conducted in 28 days, taking samples in triplicate on days 1, 7, 14, 21, and 28.

#### 2.4.2 Evaluation of the functional effect of *Bifidobacterium animalis* subsp. *lactis* BB-12

A microbiological analysis of lactic acid bacteria was performed in order to evaluate the functional effect of Petit Suisse cheese. As in the other microbiological analysis, the material was previously sterilized. The sample was prepared using a peristaltic homogenizer (Ika, USA). In addition, 1 ml of the dilution was inoculated on the 3 M™ Petrifilm™ Lactic Acid Bacteria. Later on in order to create the 10^−2^ dilution, 1 mL was transferred to a test tube containing 9 mL of sterile water. This blend was further mixed using a vortex mixer (IKA, China), and the process was repeated successively to obtain dilutions up to of 10–6. The microbiological analyses were performed in a laminar flow hood (Novatech, Mexico). For the plating methodology, the incubation took place at 48 h at 37 °C ± 1, and the counting and interpretation of the results were carried out as established by the supplier of the 3 M México plates [32]. This microbiological study was conducted for 28 days, taking samples in triplicate on days 7, 14, 21, and 28.

#### 2.4.3 Sensory analysis

**Fig. 2:**
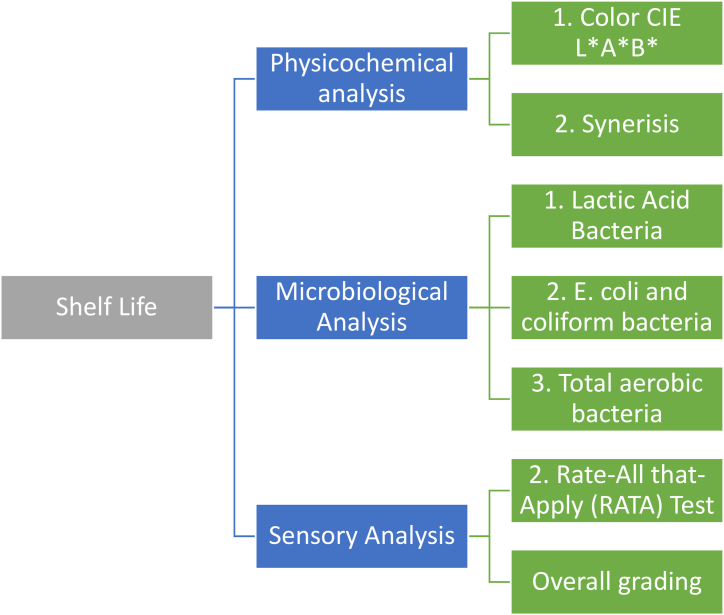
Analysis performed during Shelf Life of the guava symbiotic petit cheese product.

A sensory analysis was performed to determine the attributes that could describe the guava Petit Suisse cheese shelf life changes over time as shown in Fig. 2. Thirty semi-trained judges who had taken sensory analysis classes were invited. From these, twelve were selected and subjected to training, which consisted of selecting and defining the attributes; which were later defined according to a scale. For each analysis, eight answers were obtained.

The samples (30 g) were presented in a 1-ounce cup coded with a 3-digit number. The judges were invited to participate during the morning on days 1, 7, and 21 of the product's shelf life.

Judges were asked to try the product and answer a sensory questionnaire, which consisted of a Rate All that Apply (RATA) test, which first analyzed if an attribute was present and if it was to be rated accordingly. The following attributes were monitored: natural color, fruity odor, milky flavor, rancid flavor, bitter flavor, and firm texture.

Semi-trained judges were asked to evaluate how much they liked the product using an unstructured intensity scale, represented by a line of 11 cm that represented their beginning and end with the minimum and maximum degrees of acceptance.

### 2.5 Statistical analysis

#### 2.5.1 Physicochemical analysis

The determinations are presented as averages with standard deviations, and the analyses were conducted in triplicate.

#### 2.5.2 Organoleptic analysis

##### 2.5.2.1 Liking evaluation

This study was performed after the product was made. Data was analyzed by the system FIZZ (Software Biosystèmes, France).

##### 2.5.2.2 Sensory analysis

First, the Kolmogorov-Smirnov statistical test was used to determine if the data obtained from the level of consumer satisfaction had a normal distribution. For this test, the following hypotheses were established:

Null hypothesis (H_O_): The data obtained from the consumer's level of satisfaction are typically distributed

Alternative Hypothesis (H_1_): The data obtained from the level of consumer satisfaction are not normally distributed.

The chi-square statistical test was used as the data were not distributed normally. Since the statistical values produced by this test are known as non-parametric and distribution-free [33], it was established:

Null hypothesis (H0) that consumers like guava Petit Suisse cheese equally.

Alternative hypothesis (H_1_): Consumers do not like guava Petit Suisse cheese similarly.

##### 2.5.2.3 CATA (check all that apply)

The frequency with which consumers chose each attribute was documented for this test.

##### 2.5.2.4 Purchase intention

The frequency with which consumers decided to buy the product was documented.

#### 2.5.3 Shelf life

##### 2.5.3.1 Physicochemical analysis

Determinations are presented as average with standard deviation; analyses were performed in triplicate. Regarding the shelf life, the Fizz Software was used to analyze the sensory tests (FIZZ Software Biosystèmes, France). Through this system, a principal component analysis (PCA) was performed. PCA is a statistical test that aims to represent the variation in the data set. Visual analysis is usually presented in two or three dimensions; in this study, it is presented in two dimensions; each dimension shows the percentage of explainability that each axis has [34]. Therefore, the PCA in this study was obtained with the results from the sensory evaluation of the judges over time to evaluate the relationships between the sensory attributes. Pearson's correlation test was also used to evaluate the degree of association between sensory variables over time.

## 3 Results and discussion

### 3.1 Physicochemical analysis of elaborated product

After the triplicate determinations, the pasteurized and skimmed milk had a pH of 6.7 and an acidity of 17°D. These values aligned with the characteristics of fresh and sweet milk with a pH between 6.6 and 6.7 and a titratable acidity of 15–17°D [35]. These values are important since they indicate the microbiological quality due to the acidification of milk by acidifying bacteria, indirectly reflecting the concentration of non-fat solids, particularly proteins [36].

The guava pulp yielded a pH of 3.1, lower than that reported by Medina & Pagano [37], recorded at 4.1. This discrepancy could be attributed to the addition of citric acid, employed to impart an acid flavor and help preservation. The soluble solids were 10.05 °Brix, a value falling within the range of 9.53–11.83 °Brix obtained by Laguado et al. [38].

Inulin possibly contributed to improving the texture of the product due to its techno-functional properties, which have been reported to modify texture, which makes it a promising ingredient for dairy products. Therefore, Inulin not only helps with texture but also contributes to its prebiotic character [15,16].

While Villegas de Gante [35] indicates a cheese yield in Mexico between 9 and 11 % (total protein and casein content), which depends on the manufacturing process, our findings showed a curd yield of 17.33 %. This discrepancy may be attributed to the curd's humidity level of 78.37 % because it was neither cooked nor pressed. The curd's final composition was 3.18 % fat and 13.59 % protein in the curd. The achieved curd humidity of 78.37 % was attained through a 15-min draining period, proving conducive to the final texture of the guava Petit Suisse cheese. The pH of the curd for the current product was measured at 6.5.

### 3.2 Organoleptic analysis

**Fig. 3:**
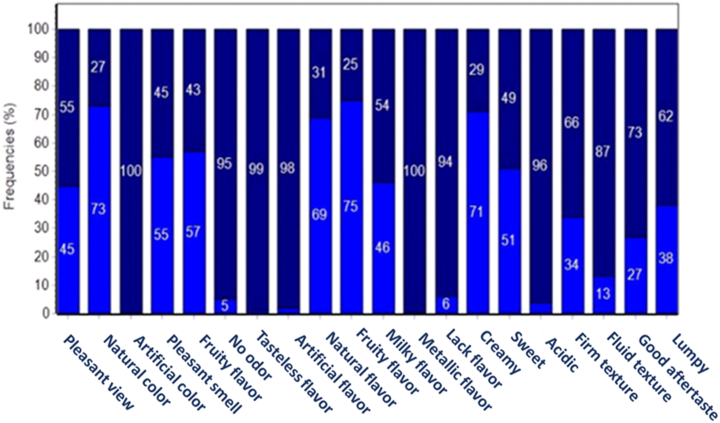
Percentage of frequencies of the sensory attributes selected by consumers in a CATA (Check all that apply) test applied to the guava symbiotic petit cheese product.

The attributes in Fig. 3 were identified by 100 consumers using the CATA (check all that apply) test. The royal blue bars correspond to the percentage of the attributes consumers select, while the dark blue bars indicate non-selected attributes. Results show that 69–75 % of consumers perceived the guava Petit Suisse cheese to have a natural, fruity, creamy flavor and natural color. Additionally, 45–57 % of consumers found the product to emit a pleasant fruity smell, impart a sweet and milky taste, and exhibit a pleasant appearance. Furthermore, 27–38 % of consumers noted a firm, lumpy texture with a good aftertaste in the guava Petit Suisse cheese. In contrast, 1–4% of consumers identified the product as sour with a lack of flavor and an artificial flavor. Notably, none of the 100 consumers detected a metallic taste or artificial color, which is significant considering that synthetic sweeteners in high concentrations contribute to a bitter metallic aftertaste [39].

On the 9-point hedonic scale, an average of 7.16 was obtained, corresponding to “I like it a lot,” with a standard deviation of 1.62.

The Kolmogorov-Smirnov statistical test yielded a value of 0.1283, at a significance level of 1 %. For which this value was compared using this value to the critical value of 0.1031 from the tables at the same significance level, the calculated value surpasses the critical value, leading us to reject the null hypothesis [40]. Since data obtained from consumer satisfaction are not typically distributed as normal data at a significance of 1 %, the chi-square statistical test was used.

The calculated chi-square value with a significance of 1 % was 37.54, surpassing the critical of 24.054 obtained from tables at the same significance level. The comparison indicates that the calculated chi-value is higher than the critical value, prompting the rejection of H_0_ and concluding that substantial evidence suggests that consumers do not equally favor the guava Petit Suisse cheese. This assertion is reinforced by 82 % of consumers' intention to buy the product from supermarkets.

### 3.3 Shelf life

The shelf life was determined based on physicochemical, microbiological, and sensory analyses. The results of the tests conducted in real-time for 28 days are presented below.

Table 2 shows a summary of the parameters analyzed. Changes begin on day 21; therefore, the product's shelf life was determined on this day. On day 28, the observed changes were more significant for all the analyzed parameters, including microbiology, color difference, and syneresis.

**Table 2:** Physicochemical and microbiological changes of guava petit cheese during shelf life.

Day	Total aerobic bacteria CFU/g	Microbiological Analysis		Color				Syneresis	Acididty
		Coliform UFC/g	*E. coli*	L*	a*	b*	ΔE*	Syneresis	acidity
1	<1	<1	0	77.1 ± 0.15	0.6 ± 0.2	12.23 ± 0.55		Not present	0.65 ± 0.01
7	<1	<1	0	77.1 ± 0.15	0.6 ± 0.2	12.23 ± 0.55	0	Not present	0.64 ± 0.02
14	<1	<1	0	76.7 ± 0.40	1.06 ± 0.11	13.1 ± 0.34	2.48 ± 1.02	Slightly syneresis	0.74 ± 0.01
21	<1	<1	0	76.4 ± 0.52	1.43 ± 0.35	11.4 ± 0.35	2.58 ± 0.0	Present	0.81 ± 0.01
28	<10	<10	0	77.2 ± 0.17	1.03 ± 0.80	13.13 ± 0.23	3.08 ± 0.42	Excessively Present	0.88 ± 0.01

Note: L*: Brightness, a*:(+) Red (−) Green, b*:(+) Yellow (−) Blue, ΔE*: Global Difference of color. *These results refer to triplicates.

#### 3.3.1 Physicochemical analysis

The guava Petit Suisse cheese was packed in transparent high-vacuum bags and refrigerated at temperatures ranging from 0 to 4 °C. Throughout the storage period, the humidity was maintained within a range of 68.05–71.47 %. This slight variation in humidity can be attributed to the release of moisture within the container, which condenses due to the temperature drop [41].

**Fig. 4:**
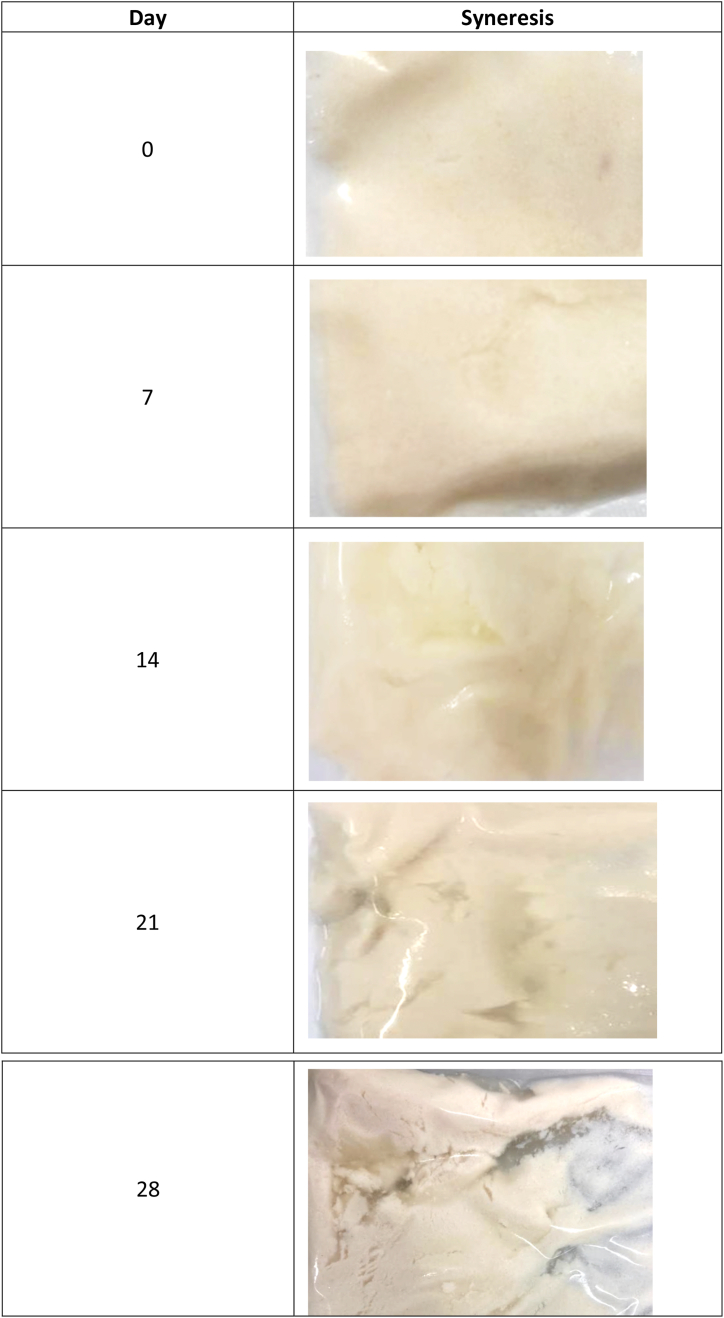
Changes in syneresis over shelf life.

Evidence of syneresis appeared slightly (Syneresis slightly present) on day 14, as shown in Fig. 4. A notable presence (Syneresis present) was observed on day 21, followed by a more pronounced manifestation (Syneresis excessively present) on day 28. Acidity has two main functions: first, it imparts flavor, and second, it is responsible for the syneresis of guava Petit Suisse cheese. The explanation for this phenomenon could be due to acidity's effect on the curd's protein network, with increased acidity correlating with heightened syneresis [42]. During the experimental process, a substantial mechanical force was applied to the curd, guava pulp, and additives using a thermomixer (Vorwerk, Germany) to obtain the characteristic texture [43]. As a result, a high syneresis was obtained due to the overall elaboration process.

The acidity in the Petit Suisse product changes significantly during storage, exhibiting discernible variations over the storage period. The statistical analysis of acidity during storage shows notable alterations, starting at 0.65 ± 0.011 and culminating at 0.88 ± 0.011 by the end of the product's shelf life.

In order to determine the color change over time in guava Petit Suisse cheese, the CIELAB color space, widely used for evaluating food color [44], was employed. Table 2 shows the results of the color coordinates. The results indicate nearly no change in the luminosity in the product (L*), attributed to little or no degradation of natural guava pigments [45]. To estimate the global color difference in the CIELAB space, the parameter ΔE* was calculated by comparing the product's color on day 0 with samples taken over 28 days. As observed in Table 2, the overall color difference increased as time progressed, aligning with the transition from yellow to subtly red hues in the product's color.

#### 3.3.2 Microbiologic analysis

Table 2 shows the cfu/g of total bacterial counts, coliforms, and *E. coli*. It can be observed from the table that there was no growth of mesophilic aerobic bacteria during the 21 days. Total aerobic count reflects the sanitary quality of food, handling practices, and the hygienic conditions of raw materials [46]. The growth appeared at 28 days, with a count of 10 cfu/g.

The behavior of total coliforms mirrored that of aerobic mesophilic bacteria. No growth was observed during the initial 21 days, with a count of 10 cfu/g on day 28 (Table 2). These bacteria are typically found in the intestine of humans and animals, as well as soil, seeds, and vegetables. Their presence does not necessarily indicate fecal contamination or pose a health risk [47]. Total coliforms provide insights into the cleanliness, disinfection processes, and microbiological quality of water, vegetables, and processed products. Throughout the shelf life study, *E. coli* was absent, in compliance with the maximum permissible limit of 100 cfu/g established by NOM-243-SSA1-2010 [26].

#### 3.3.3 Viable bacterial count *Bifidobacterium animalis* subsp. *lactis* BB-12

**Fig. 5:**
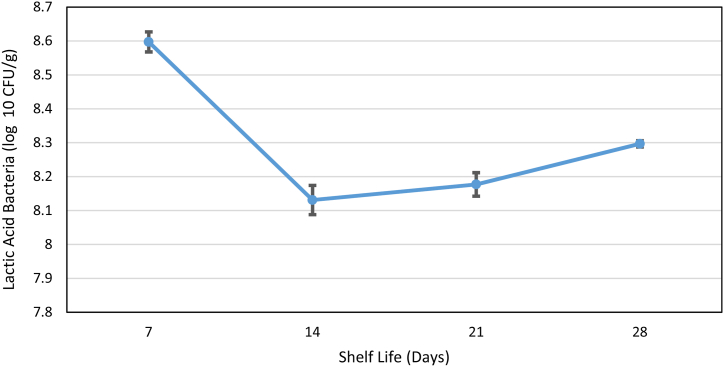
Concentration of lactic acid bacteria during shelf life of the guava symbiotic petit cheese product.

The added concentration of this probiotic was 0.2 %, resulting in a finished product concentration exceeding 1 × 10^6^ CFU/g to exert clinical and therapeutic effects on consumers [48,49]. The viable lactic acid bacteria count began at 8.59 log10 CFU/g, equivalent to 3.96 × 10^8^ CFU/g on day seven. This count slightly decreased over the days, changing to 1.98 × 10^8^ CFU/g to 8.29 log10 CFU/g on day 28 (Fig. 5). Therefore, the probiotic functionality of guava Petit Suisse cheese, as the microbial concentration must be equal to or surpass 1 × 10^6^ CFU/g and must remain viable at the time of consumption to exert its clinical and therapeutic effects [48,49]. The obtained count of lactic acid bacteria corresponds to the growth of the probiotic *Bifidobacterium animalis* subsp growth. *lactis* BB-12, as indicated by medium acidification of the absence of other bacterial growth.

#### 3.3.4 Sensory analysis

**Fig. 6:**
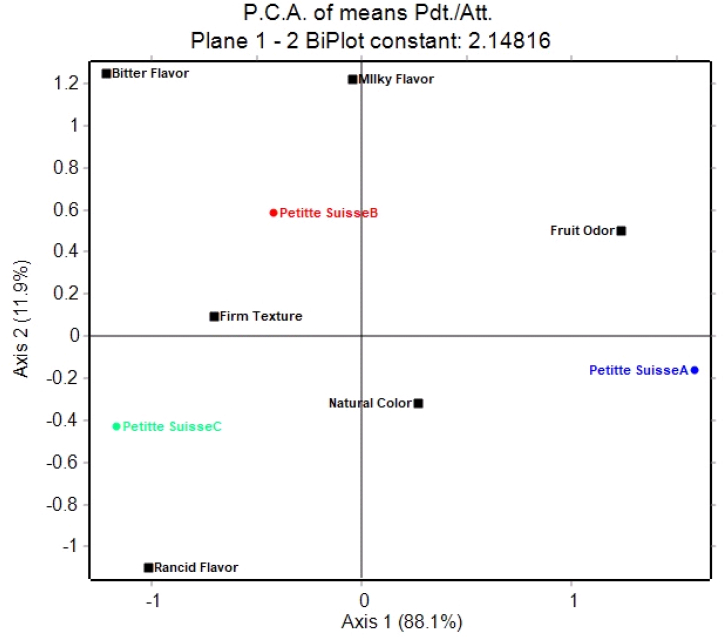
Changes sensory characteristics during shelf life using a PCA (Principal Component Analysis) for the guava symbiotic petit cheese product (Petitte Suisse A = first week of storage; Petitte Suisse B: second week of storage and Petitte Suisse C: third week of storage).

Fig. 6 shows the principal component analysis (PCA) performed with the sensory analysis results throughout the shelf life. The model can be explained by this PCA 100 %, with the first component representing 88.1 % of the total information, while the second component represents 11.9 %; which means that the explainability of the model is 100 %. This is a different way to express how the key sensory descriptors change regarding time. Petite Suisse A (day 7), B (day 14), and C (day 21), represent the shelf life. These analyses reveal the interrelation of sensory variables throughout shelf life. For instance, natural color and fruity smell can be appreciated on day 7 (petite cheese A). In contrast, on day 14 (petite cheese B), the lactic flavor emerges alongside a bitter taste. Finally, by day 21 (petite Suisse C), a change in texture is accompanied by the perception of a rancid taste.

It is important to notice that bitter taste, rancid taste, fruity odor, and milky taste, appear farthest from the origin. Conversely, descriptors like natural color and firm texture are considered less influential. Bitter taste and lactic flavor exhibit a strong correlation, while fruity odor displays an inverse relationship with rancid flavor, remaining independent of other descriptors. Furthermore, natural color is inversely correlated with bitter taste.

In order to evaluate the degree of association between sensory variables over time, a Pearson correlation was performed, indicating covariation between at least two variables in a linear correlation [50]. Significant correlations were identified through a statistically evaluated correlation analysis with a probability ≤0.05, as outlined in Table 3. The variables fruity smell - dairy taste and rancid taste - bitter taste presented a moderate relationship with correlation coefficients of 0.5955 and 0.5845, respectively. The relationship between natural colors - and dairy flavor resulted in the least significant relationship coefficient of 0.3734. Since all correlation coefficients are positive, the variables increase in tandem [32], indicating that rancid and bitter tastes increase with time.

**Table 3:** Significant Pearson correlations with a significance of 5 % regarding attributes of.

Attribute 1	Attribute 2	Correlation coefficient	Probability
fruity smell	dairy flavor	0.5955	0.0007
rancid taste	bitter taste	0.5845	0.0009
color Natural	fruity smell	0.4392	0.0171
dairy flavor	bitter taste	0.4006	0.0313
color Natural	dairy flavor	0.3734	0.0460

The natural color perception decreased until day 21, possibly due to the degradation of the natural pigments in the guava. A study by Ventosa et al. [51] identified and quantified phytofluene, β-carotene, and lycopene as major carotenoids in guava *(Psidium guajava* L*).* Packaging the Petit Suisse cheese in transparent vacuum bags could affect carotenoid stability, as light can lead to their breakdown and the formation of colorless, low molecular weight compounds [52]. A pH decrease and increased acidity could also influence color due to carotenoid cis/trans isomerization, rearrangements, and de-esterification [52].

The fruity odor decreased over 21 days, which could be attributed to the loss of low molecular weight and highly volatile aroma compounds generated by lipid oxidation [38].

The rancid taste increased considerably on day 21. The changes could be due to the biochemical process called lipolysis, a biochemical process transforming triglycerides into partial glycerides and free fatty acids [53]. Free fatty acids with carbon atoms ranging from 4 to 12 can generate rancid aromas and flavors [54,55].

The bitter taste increased during the 21 days, particularly in the third week. This phenomenon could be linked to the proteolysis generated in fresh cheeses characterized by high humidity and low pH, resulting in an excessively soft texture and bitter taste [56].

On day 21, the milk flavor decreased, likely due to lipolysis and proteolysis reactions generating alternative flavors and aromas, altering the original milk flavor. Other factors, such as environmental conditions, microorganisms, or compound oxidation, could also influence milk flavor [57].

The texture changed over the three-week period, potentially attributed to enzymatic activity attacking the protein matrix, altering sensory properties such as color, elasticity, and cheese texture [58,59].

## 4 Conclusions

A guava functional symbiotic petit cheese product was successfully developed, incorporating probiotics (*Bifidobacterium animalis* subsp. *lactis* BB-12, Chr. Hansen, Denmark) and prebiotics (Inulin) in a dairy matrix. The product underwent a comprehensive organoleptic evaluation, revealing noteworthy results. Participants rated the product on a 9-point scale, yielding an average of 7.16, corresponding to a strong preference (“I like it a lot”), with a standard deviation 1.62.

Attributes identified by consumers highlighted positive perceptions of the product: 69–75 % noted a natural, fruity, and creamy flavor with a natural color. Furthermore, 45–57 % recognized a pleasant fruity smell, a sweet and milky taste, and a pleasant appearance. In addition, 27–38 % of consumers found a firm, lumpy texture with a good aftertaste in guava Petit Suisse cheese. In contrast, a minimal percentage of 1–4% described the product as sour or lacking flavor, with an artificial flavor. Encouragingly, none of the consumers found an artificial color or metallic taste. Impressively, 82 % of participants expressed their intent to buy the product.

The physicochemical and sensory characteristics of the final product were systematically monitored during its shelf life. Notably, real-time shelf life assessment demonstrated that syneresis increased on day 21, marking the critical parameter defining the product's shelf life. Meanwhile, humidity exhibited negligible fluctuations. Colorimetric analysis revealed changes in the global difference in color. Sensory evaluations across the shelf life indicated that the lactic flavor and texture remained stable. However, certain changes were observed: the natural color decreased due to the degradation of carotenoids under the influence of light and decreasing pH. Fruity odor reduction was attributed to the loss of volatile compounds from the guava. The bitter and rancid tastes intensified over time, potentially due to lipolysis and proteolysis reactions. By day 21, semi-trained judges assigned an average overall liking score of six on a scale from 1 to 10.

Microbiological analysis showed that coliforms and total aerobic bacteria grew after day 28 without surpassing established limits, ensuring product safety. Notably, no presence of *E. coli* was detected, guaranteeing an innocuous product. The analysis of lactic acid bacteria indicated viable *Bifidobacterium animalis* subsp. *lactis* BB12 presence, with a concentration of 1.98 × 10^8^ CFU/g at 28 days. Therefore, the results can confirm the functional effect of the product, as the minimum required concentration must be 1 × 10^6^ CFU/g. Ultimately, the product's shelf life was determined to be 21 days due to the presence of syneresis at this day.

The study's strengths rely on the project's well-defined objective: to develop a functional guava petit cheese that consumers would accept. Moreover, this product could be identified as a value-added dairy product with a healthy character due to the incorporation of probiotics and prebiotics and the particular sensory attributes, specifically the guava flavor. This project used robust research methods detailed in this paper's procedures. Upon industrial-scale trials, this product could be ready for commercialization. Moreover, the nature of the product will allow the incorporation of other natural ingredients to improve the diversity of Petit Suisse products in Mexico and Latin America. Moreover, the product could be marketed into specific segments, for example, adults. On the other hand, an area of opportunity for this study could be the calculation of the shelf-life using the Arrhenius model.

## Ethic statement

Consumers signed a consent form digitally. On the other hand, 10.13039/100016992Universidad Panamericana Aguascalientes approved this study through the study number DNA/ING/2023/4.

## Use of IA

During the preparation of this work, the author(s) used Chat GPT for translation purposes. The translation was from Spanish to English to improve the quality of the research paper. Later, the authors used Grammarly to check the document's style. After using these tools/services, the author(s) reviewed and edited the content as needed. Therefore, we take(s) full responsibility for the publication's content.

## Research data for this article

The raw data required to reproduce the above findings are available on request to the corresponding author.

## CRediT authorship contribution statement

**Victor Iván Morales-Cortés:** Formal analysis, Data curation, Conceptualization. **Julieta Domínguez-Soberanes:** Writing – review & editing, Writing – original draft, Visualization, Validation, Supervision, Software, Resources, Project administration, Investigation, Funding acquisition, Formal analysis, Data curation, Conceptualization. **Linda Carolina Hernández-Lozano:** Visualization, Validation, Supervision, Project administration, Methodology, Investigation. **Carmen C. Licon:** Writing – original draft, Methodology, Investigation, Conceptualization. **Antonio Estevez-Rioja A:** Methodology, Data curation. **Mayeli Peralta-Contreras:** Methodology, Data curation.

## Declaration of competing interest

All the authors participated in the development and design of the work and we believe that the manuscript represents valid work, carefully read and fully approved. Moreover, we would like to indicate that there is no bias.

So we would like to state a Declaration of interest: None
