# Supplementary material for: Sensory characterization of functional guava symbiotic petit cheese product
Source: Heliyon. 2023 Nov 7;9(11):e21747. doi: 10.1016/j.heliyon.2023.e21747 (PMC10681930; doi:10.1016/j.heliyon.2023.e21747)

Questionnaires applied

Two sensory questionnaires applied during the research using a FIZZ (Software Biosystèmes, France). This works as an app in which the information is presented through different screens and consumers and judges have to select the answers.

1. Sensory Characterization of the New Developed Product (Consumers)

The first questionnaires had different sections.

- Firstly, an acceptability test was applied, in order to know how much was the product liked.
- Secondly, a CATA (Check all that apply) in order to define a profile for the new developed product.
- Thirdly, the purchase intention was evaluated. For which 100 consumers were instructed to taste, the product ant select the attributes that best define the product.

1. **How much did you like the product?**

| 9 | Like Extremely |
| --- | --- |
| 8 | Like Very Much |
| 7 | Like Moderately |
| 6 | Like Slightly |
| 5 | Neither Like nor Dislike |
| 4 | Dislike Slightly |
| 3 | Dislike Moderately |
| 2 | Dislike Very Much |
| 1 | Dislkike Extremely |

1. **Check all the attributes that best define the product (minimum 10).**

fruity taste

milky taste

rancid flavor

lack of taste

creamy

sweet

acid

firm texture

fluid texture

good aftertaste lumpy.

pleasant appearance

natural color artificial color pleasant smell fruity smell

bland taste artificial taste natural taste

1. **Would you buy this product at the supermarket?**

Yes No

1. Shelf life of the the guava petit Suisse

The questionnaire applied to the guava-flavored petit suisse cheese for the Shelf life of the product underwent a RATA (Rate all that apply) questionnaire and an overall liking. For which, twelve semi-trained judges were invited.

1. **Please rate the intensity of the following attributes based on the sample of petit Suisse sample:**

Natural Color

Not at all

A lot


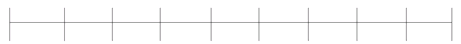


Fruity Odor

Not at all

A lot


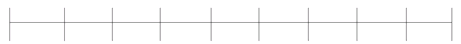


Milky Flavor

Not at all

Nada

A lot


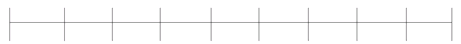


Rancid flavor

Not at all

Nada

A lot


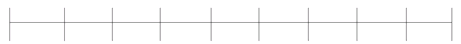


Bitter flavor

Not at all

Nada

A lot


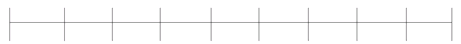


Firm Texture

Not at all

Nada

A lot


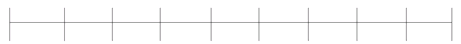


**2. How much did you like the product?**


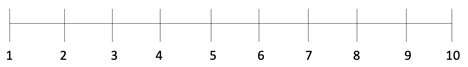

Supplement: Multimedia component 1 [file mmc1.docx]
